# The Assessment of the Prevalence and Disability Severity of Musculoskeletal Pain in Patients With Multiple Sclerosis in Saudi Arabia

**DOI:** 10.7759/cureus.32413

**Published:** 2022-12-11

**Authors:** Khaled A Amer, Abdulrahman A Aldosari, Mansour Y Somaily, Rammas A Shawkhan, Razan A Almuhsini, Mohammed A Al Mater, Aseel I Al Saleh, Maram S Alshabeeb, Fahad S Alshahrani

**Affiliations:** 1 College of Medicine, King Khalid University, Abha, SAU; 2 Department of Medicine, Rheumatology Division, King Khalid University Medical City, Abha, SAU; 3 Department of Medicine, Section of General Medicine, Asir Central Hospital, Abha, SAU

**Keywords:** saudi arabia, determinants, prevalence, pain, musculoskeletal disorders, multiple sclerosis

## Abstract

Background

Multiple sclerosis (MS) is an autoimmune disease of the nervous system that causes chronic demyelination over time and may lead to physical disability. MS-related pain may be musculoskeletal, paroxysmal, or persistently neurogenic in nature. The most common type of pain is musculoskeletal discomfort, which is typically brought on by muscle weakening, stiffness, and generalized imbalance as the condition progresses. Pain often manifests after prolonged immobilization of muscles, tendons, and ligaments.

Aim

We aimed to evaluate the prevalence and severity of musculoskeletal pain (MSP) among MS patients in Saudi Arabia.

Methodology

A quantitative cross-sectional study was conducted. Patients with confirmed MS in Saudi Arabia were invited to participate in the study during the duration from April 2022 to May 2022. Data were collected using an electronic collection tool. The study tool was checked to ensure the content validity and clarity of the Arabic and English versions.

Results

A total of 360 MS patients were included. Patients’ ages ranged from 18 to 65 years with a mean age of 34.9±13.2 years old. Exactly 229 (63.6%) patients were females. A total of 104 (28.9%) patients complained of relapsing-remitting MS, 34 (9.4%) complained of primary progressive MS, and 16 (4.4%) complained of secondary progressive MS. A total of 138 (38.3%) patients had the disease for less than five years, and 14 (3.9%) had the disease for more than 21 years. Exactly 124 (34.4%) MS patients complained of high disability due to MSP, while 236 (65.6%) had low disability.

Conclusions

This study demonstrates that one out of each three patients with MS complained of pain with high disability associated with pain. Old age, comorbidities, long disease duration, and a family history of MS were significant determinants of associated disability severity.

## Introduction

Multiple sclerosis (MS) is an autoimmune disease of the nervous system that causes chronic demyelination over time and may lead to physical disability [1]. About 350,000 individuals in the USA and millions around the world have been diagnosed with MS, which is considered to be the most common nontraumatic cause of disability in young adults [2,3]. Pain is the usual complaint with many other symptoms and difficulties [4]. According to the literature, the prevalence of pain in MS patients ranged from 29% to 86%, and 23% of MS patients report to have suffered from pain at the time of diagnosis [5-7].

Symptoms of pain usually associated with MS include musculoskeletal, paroxysmal, or persistent neurogenic pain [8]. The most common type of pain is musculoskeletal discomfort, which is brought on by the disease’s progression and muscle weakening, stiffness, and general imbalance. When tendons, ligaments, and muscles remain immobilized for an extended period of time, pain typically develops [9]. Weakness and spasticity can result in painful contractures that may be severe as prescribed by MS patients [10].

In addition to the wide range of reported prevalence and associated factors with pain in MS patients, neuropathic pain was the focus of the majority of investigations. A case series investigation found that dimethyl fumarate, which is routinely prescribed to people with multiple sclerosis, may have an uncommon musculoskeletal pain (MSP) side effect [11]. The prevalence, nature, and causes of MSP in MS disease are not well understood, however. Therefore, the purpose of this study was to examine the prevalence, kind, and distribution of pain and related impairment among Saudi Arabian patients with confirmed MS diagnoses.

## Materials and methods

Study design and subjects

A quantitative cross-sectional study was conducted. Patients with confirmed MS in Saudi Arabia were invited to participate in the study with the help of patient support groups for MS in Saudi Arabia during the duration from April 2022 to May 2022. Any patient diagnosed with MS for more than one year, aged more than 18, and living in Saudi Arabia was included. Unconfirmed MS-diagnosed or suspected cases were excluded.

Data collection tool and research instrument

Google Forms (Google, Inc., Mountain View, CA, USA) using a self-administered questionnaire connected to an Excel sheet (Microsoft Corp., Redmond, WA, USA) was used for data collection, which consisted of demographic data, questions related to MS type and treatment, and the Örebro Musculoskeletal Pain Questionnaire (ÖMPQ) [12]. The study questionnaire was checked to ensure the content validity and clarity of the Arabic and English versions.

Data analysis

Data were revised, coded, and fed into the Statistical Package for the Social Sciences (SPSS) software (version 23 for Mac) (IBM SPSS Statistics, Armonk, NY, USA). Two-tailed tests were used for analysis. P<0.05 was considered statistically significant. Regarding MS-associated and MSP-associated disability, the Örebro Musculoskeletal Pain Questionnaire was scored with reference to reported scoring methods; then, the overall score was obtained by summing up all items circled score level (out of 10). The total score will range between 1 and 100, with a score of >50 indicating a higher estimated risk for disability [12]. All variables, including biodemographic information, clinical type, duration, and medications taken by MS patients, and the improvement, were applied to a descriptive analysis based on frequency and percent distribution. Also, patients’ overall MSP-associated disability level was graphed. Cross-tabulation was used to assess the relation between MSP-associated disability levels by MS patients’ biodemographic data and MS clinical data. Pearson’s chi-squared test and an exact probability test for small frequency distributions were used to examine the relationship between variables.

Ethical approval

Each participant voluntarily participated after being informed of the study’s aims. The study was approved by the Aseer Institutional Review Board, Directorate Health Affairs, Aseer Region, with institutional review board (IRB) registration number H-06-B-091.

## Results

A total of 360 MS patients were included. Patients’ ages ranged from 18 to 65 years with a mean age of 34.9±13.2 years old. Exactly 229 (63.6%) patients were females, and 275 (76.4%) were university graduates, while 79 (21.9%) had a secondary level of education or diploma. A total of 174 (48.3%) patients were married, and 152 (42.2%) were single. As for work, 222 (61.7%) patients were working, and 81 (22.5%) were not. Exactly 6.4% of the patients complained of thyroid disorders, 3.6% were hypertensive, 3.1% were diabetic, and 75.3% had no other comorbidities. Also, 21 (5.8%) of the study patients had other autoimmune diseases, and 55 (15.3%) had a family history of MS, as shown in Table 1.

**Table 1:** Biodemographic data of the study patients with multiple sclerosis disorder MS: multiple sclerosis, DM: diabetes mellitus, HTN: hypertension

Biodemographic data	Number	%
Age in years		
<30	139	38.6%
31-40	154	42.8%
41-50	48	13.3%
>50	19	5.3%
Gender		
Male	131	36.4%
Female	229	63.6%
Educational level		
Below secondary	6	1.7%
Diploma/secondary	79	21.9%
University/above	275	76.4%
Marital status		
Single	152	42.2%
Married	174	48.3%
Divorced/widow	34	9.4%
Work		
Not working	81	22.5%
Student	38	10.6%
Working	222	61.7%
Retired	19	5.3%
Other comorbidities		
None	271	75.3%
DM	11	3.1%
HTN	13	3.6%
Thyroid disorder	23	6.4%
Others	42	11.7%
Have another autoimmune disease		
Yes	21	5.8%
No	339	94.2%
Family history of MS		
Yes	55	15.3%
No	268	74.4%
Do not know	37	10.3%

Table 2 shows MS clinical data and treatment among study cases. Regarding the type of MS, 104 (28.9%) complained of relapsing-remitting MS, 34 (9.4%) complained of primary progressive MS, and 16 (4.4%) complained of secondary progressive MS. A total of 138 (38.3%) patients had the disease for less than five years, and 14 (3.9%) had the disease for more than 21 years. Exactly 207 (57.5%) received only one drug, 51 (14.2%) received more than two drugs, and 54 (15%) had no drugs. A total of 89 (29.1%) only used medication to reduce seizures caused by MS, and 122 (39.9%) felt an improvement in their symptoms when they take MS medications.

**Table 2:** Multiple sclerosis clinical data and treatment among the study cases MS: multiple sclerosis

MS clinical data	Number	%
Type of MS disorder		
Relapsing-remitting MS	104	28.9%
Primary progressive MS	34	9.4%
Secondary progressive MS	16	4.4%
Do not know	206	57.2%
Duration of the disease (years)		
<5	138	38.3%
5-9	91	25.3%
10-14	82	22.8%
15-21	35	9.7%
>21	14	3.9%
Number of treatments received		
One drug	207	57.5%
Two drugs	48	13.3%
>2 drugs	51	14.2%
No drugs	54	15%
Do you use any medication to reduce seizures caused by multiple sclerosis?		
Yes	89	29.1%
No	217	70.9%
Do you feel an improvement in your symptoms when you take multiple sclerosis medications?		
Yes	122	39.9%
No	184	60.1%

**Figure 1:**
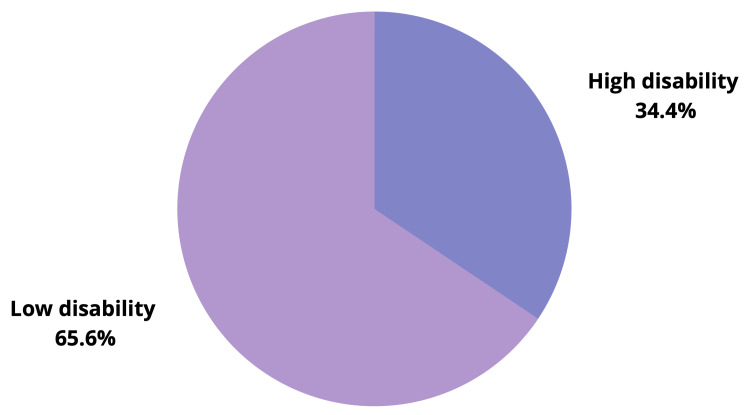
Severity of musculoskeletal pain and associated disability among the study participants

Figure 1 shows that 124 (34.4%) MS patients complained of high disability due to MSP, while 236 (65.6%) had low disability.

Table 3 demonstrates that 63.2% of MS patients aged more than 50 years complained of high disability due to MSP compared to 26.6% of others aged less than 30 years with recorded statistical significance (P=0.001). Also, 89.5% of retired patients had high disability versus 39.5% of those who were not working and 29.7% of working cases (P=0.001). Of the hypertensive MS patients, 61.5% had high disability in comparison to 29.9% of those with no other comorbidity (P=0.023).

**Table 3:** Distribution of musculoskeletal disability level by patients’ biodemographic data P-values are calculated using Pearson’s chi-squared test. ^$^Exact probability test *P<0.05 (significant) MSK: musculoskeletal, MS: multiple sclerosis, DM: diabetes mellitus, HTN: hypertension

Biodemographic data	MSK disability level				P-value
	Low disability		High disability		
	Number	%	Number	%	
Age in years					0.001*
<30	102	73.4%	37	26.6%	
31-40	105	68.2%	49	31.8%	
41-50	22	45.8%	26	54.2%	
>50	7	36.8%	12	63.2%	
Gender					0.342
Male	90	68.7%	41	31.3%	
Female	146	63.8%	83	36.2%	
Educational level					0.293^$^
Below secondary	3	50%	3	50%	
Diploma/secondary	47	59.5%	32	40.5%	
University/above	186	67.6%	89	32.4%	
Marital status					0.061
Single	109	71.7%	43	28.3%	
Married	109	62.6%	65	37.4%	
Divorced/widow	18	52.9%	16	47.1%	
Work					0.001*
Not working	49	60.5%	32	39.5%	
Student	29	76.3%	9	23.7%	
Working	156	70.3%	66	29.7%	
Retired	2	10.5%	17	89.5%	
Other comorbidities					0.023*^$^
None	190	70.1%	81	29.9%	
DM	6	54.5%	5	45.5%	
HTN	5	38.5%	8	61.5%	
Thyroid disorder	12	52.2%	11	47.8%	
Cardiac	0	0%	0	0%	
Others	23	54.8%	19	45.2%	
Have another autoimmune disease					0.912
Yes	14	66.7%	7	33.3%	
No	222	65.5%	117	34.5%	
Family history of MS					0.411
Yes	32	58.2%	23	41.8%	
No	178	66.4%	90	33.6%	
Do not know	26	70.3%	11	29.7%	

Table 4 shows that 50% of MS patients with long disease duration (>21 years) had high disability compared to 26.8% of recently diagnosed cases (P=0.008). Also, high disability was detected among 43.1% of MS patients on more than two drugs versus 29.5% of others who had only one drug for MS (P=0.049). Additionally, 47.2% of patients who used medication to reduce seizures caused by MS had high disability compared to 29.5% of others who did not (P=0.003). Also, high disability was detected among 40.2% of MS without a family history of the disease versus 26.2% of others with (P=0.012).

**Table 4:** Distribution of musculoskeletal disability level by patients’ multiple sclerosis-related data P-values are calculated using Pearson’s chi-squared test. ^$^Exact probability test *P<0.05 (significant) MSK: musculoskeletal, MS: multiple sclerosis, DM: diabetes mellitus, HTN: hypertension

MS clinical data	MSK disability level				P-value
	Low disability		High disability		
	Number	%	Number	%	
Type of MS disorder					0.216
Relapsing-remitting MS	72	69.2%	32	30.8%	
Primary progressive MS	24	70.6%	10	29.4%	
Secondary progressive MS	7	43.8%	9	56.3%	
Do not know	133	64.6%	73	35.4%	
Duration of the disease (years)					0.008*
<5	101	73.2%	37	26.8%	
5-9	65	71.4%	26	28.6%	
10-14	45	54.9%	37	45.1%	
15-21	18	51.4%	17	48.6%	
>21	7	50%	7	50%	
Number of treatments received					0.049*
One drug	146	70.5%	61	29.5%	
Two drugs	25	52.1%	23	47.9%	
>2 drugs	29	56.9%	22	43.1%	
No drugs	36	66.7%	18	33.3%	
Do you use any medication to reduce seizures caused by multiple sclerosis?					0.003*
Yes	47	52.8%	42	47.2%	
No	153	70.5%	64	29.5%	
Do you feel an improvement in your symptoms when you take multiple sclerosis medications?					0.012*
Yes	90	73.8%	32	26.2%	
No	110	59.8%	74	40.2%	

## Discussion

MS is an autoimmune condition that affects the central nervous system and is characterized by chronic demyelination over time and may result in physical impairment. The documented disease incidence is 2.5 per 100,000 people worldwide, and there are 2.5 million instances of it [13-15]. About three-quarters of MS patients report having experienced pain in the month prior, and nearly half of MS patients reported experiencing pain [16]. Age, disease duration, disease progression, and patient physical impairment were the most often reported factors linked with pain severity, according to the literature [17].

Many studies in the literature show a high prevalence of MSP among MS patients. However, no study was conducted in Saudi Arabia on whether MS patients experiencing MSP need to be studied besides its associated risk factors and which patients are more prone to it. The treatment of MSP among patients with MS differs based on the severity of the pain and the classification of MS. The treatment could be in a pharmacological or physiotherapy form.

This study demonstrates that about one-third of MS patients (34.4%) complained of high disability due to MSP, while the other two-thirds had low disability with little pain-related effect on their daily life and work. Regarding factors associated with MSP and disability, the study showed that old aged patients, retired/not working patients, and patients with comorbidities including hypertension and diabetes mellitus were the main biodemographic factors. Regarding patients’ MS data, the study showed that long disease duration and receiving more than two drugs, which indicates high progression of MS disorder, were the most significant determinants for high disability among the study cases.

About 55.6% of MS patients reported MSP, while 21.1% reported neuropathic pain. Age, gender, disease duration, employment position, marital status, and education level did not significantly differ between the studied group. The Expanded Disability Status Scale (EDSS) ratings of the participants with MSP were lower than those of the participants with neuropathic pain [18]. Solaro et al. [5] revealed that 43% of MS patients experienced pain, which was substantially correlated with age, disability, disease duration, and disease course. Similar results were shown by another study [19]. In the study of Massot et al. [20], the median EDSS score was 6.

A secondary progressive form of MS affected about half of the patients, while a relapse-remitting form affected 27.4% of the studied cases. The most prevalent musculoskeletal conditions were genu recurvatum, claw toe, and knee osteoarthritis. Shayesteh-Azar et al. [21], in their study, revealed that 87.8% of MS patients reported experiencing pain at some point during the study. The average pain level was 3.75±2.25, while the severe pain was 5.73±2.12. The areas with the highest levels of pain were the knees (55.7%), wrists (43.5%), and neck (41.7%). Ankle, upper back, and shoulder pain seem to be more common in women. In more than half of MS cases, daily functioning was restricted at least temporarily. In cases with shorter disease duration, upper back and neck pain was more frequent.

Svendsen et al. [22] observed that patients had pain in many limbs at once. Additionally, they noted that the lower limbs (96%), back (70%), and upper limbs (52%) were the most severely affected. Osterberg et al. [23] revealed in their study that lower limb pain was reported to be 87% common and upper limb pain to be 31%.

This study is distinguished by the use of unique factors that have not been researched in Saudi Arabia before. However, there are several restrictions on our study that must be addressed. Even if we employ a legitimate and trustworthy technique to gather data, the cross-sectional nature of the study and the use of the electronic survey as the main data collection method may make the study findings vulnerable to recall bias. Potential confounders could not be explicitly ruled out. Further study with a stronger study design needs to be conducted to ensure the causal relationship.

## Conclusions

This study demonstrates that one out of three patients with MS complained of pain with high disability associated with pain. Old age, comorbidities, long disease duration, and a family history of MS were significant determinants of associated disability severity. Periodic surveys and health education are recommended for identifying and introducing reasonable accommodations that can enable MS patients with disabilities to find and retain their ordinary life with the lowest effect on their quality of life.
